# Benign Intracranial Hypertension: A Rare Manifestation of Neurosarcoidosis

**DOI:** 10.7759/cureus.43363

**Published:** 2023-08-12

**Authors:** Prashant Dubey, Satish Nirhale, Shalesh Rohatgi, Pranit Khandait

**Affiliations:** 1 Department of Neurology, Dr. D.Y. (Dnyandeo Yashwantrao) Patil Medical College, Hospital & Research Centre, Pune, IND

**Keywords:** hypothyroidism, bell's palsy, idiopathic intracranial hypertension (iih), neurosarcoidosis, granulomatous inflammation, pseudotumor cerebri, benign intracranial hypertension

## Abstract

Sarcoidosis is an immune-mediated disease that can involve multiple systems. Sarcoidosis of the nervous system or neurosarcoidosis may present as cranial mononeuropathy, hypothalamic involvement, aseptic meningitis, granulomatous inflammation in the brain parenchyma or spinal cord, peripheral neuropathy, and, in rare cases, as myopathy and benign intracranial hypertension. The most common cranial nerve involvement is the facial nerve, which can present as unilateral or bilateral facial nerve palsy, often with recurrent episodes. Involvement of other cranial nerves such as the second and eighth cranial nerves has also been reported. Granulomatous inflammation in the spinal cord presents as myelopathy or radiculopathy. Peripheral neuropathy can manifest as mononeuropathy, mononeuritis multiplex, or generalized sensory-motor neuropathy. Carpal tunnel syndrome is more common in patients with sarcoidosis compared to the general population. Here, we describe the case of a 40-year-old female who presented with heaviness of the head and blurred vision, with a prior history of left-sided Bell's palsy. Bilateral papilledema was observed during the fundus examination. MRI of the brain revealed signs suggestive of benign intracranial hypertension. The cerebrospinal fluid (CSF) opening pressure was measured at 40 cmH2O. Biopsy of bilateral hilar lymphadenopathy indicated granulomatous inflammation consistent with sarcoidosis. The patient was started on steroids and acetazolamide, and she had a dramatic improvement in symptoms.

## Introduction

Sarcoidosis was first described in the 19th century with the involvement of one or more systems [1]. Nervous system involvement is a very rare presentation and requires a high index of suspicion. It most commonly manifests as recurrent facial nerve palsy [2-6]. Early recognition is important to initiate prompt therapy, and sarcoidosis responds well to steroids [3]. The long-term outcome is good in neurosarcoidosis, as its autoimmune process is composed of T-cell, CD4 positivity, macrophages, epithelioid cells, and multinucleated giant cells form sarcoid granuloma. To halt this autoimmune process, long-term immunosuppression is required [2].

Here, we describe the case of a 40-year-old female presenting with benign intracranial hypertension and a history of left-sided Bell's palsy. The patient also had hilar lymphadenopathy, along with a high serum angiotensin-converting enzyme (ACE) level. Hilar lymph node biopsy confirmed the diagnosis of sarcoidosis.

## Case presentation

A 40-year-old female presented with a heaviness of the head for the past 15 days, accompanied by a gradual progressive diminution of vision in both eyes over the past 10 days. The patient reported a history of transient visual obscuration for the past eight days, specifically when she got up from bed. The patient experienced episodes of grey-outs, black-outs, or blur-outs of vision lasting for three to five seconds. but denied any history of double vision. Three months prior to this presentation, she had a history of left-sided Bell's palsy with partial recovery. There was no history of other cranial nerve involvement, motor or sensory system involvement, seizures, recent fever or vaccination, oral contraceptive pill intake, vitamin supplementation, or weight gain. Her BMI was 23.3 kg/m². She was a known case of type 2 diabetes mellitus and hypothyroidism, both of which were well controlled with medication. There were no recent modifications in her medication over the past six months.

**Figure 1:**
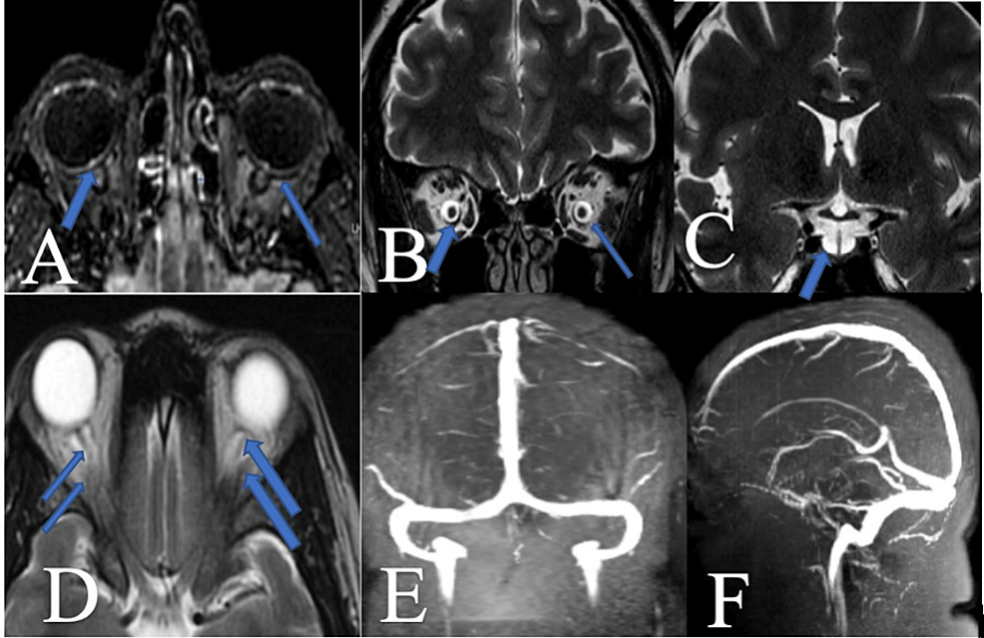
MRI brain and venogram (A) Fluid-attenuated inversion recovery (FLAIR) sequence showed hyperintensity at the optic disc with posterior globe of orbit flattening; (B) T2-weighted (T2W) coronal dilatation of optic nerve sheet; (C) T2-weighted (T2W) coronal dilatation of empty sella turcica; (D) T2W axial orbital cuts showed tortuosity of optic nerve; (E, F) Normal dural venous sinus system

**Figure 2:**
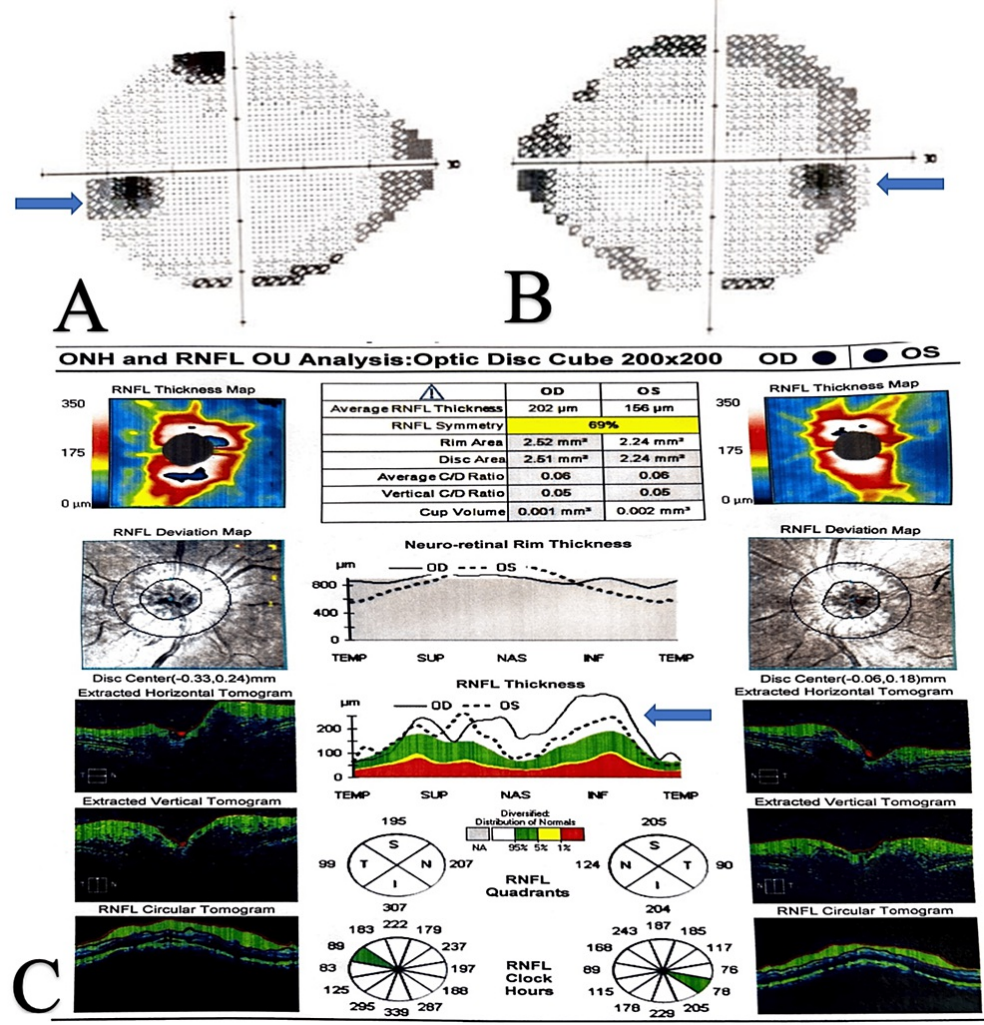
(A) Perimetry of left eye and (B) perimetry of the right eye showed enlargement of blind spot with constriction of peripheral field; (C) Optical coherence tomography revealed bilateral disc oedema ONH: optic nerve hypoplasia; RNFL: retinal nerve fiber layer; OU: oculus uterque (both eyes); OS: oculus sinister (left eye); OD: oculus dexter (right eye)

On examination, her vital parameters were within normal limits, and bilateral papilledema (Grade I) was observed. Blurring of vision beyond a distance of 5 feet was noted in both eyes. Residual weakness of the left-sided Bell's palsy was present (HouseBrackmann facial paralysis grade-3). The rest of the neurological examination was within normal limits. Routine blood laboratory investigations, including serological markers and thyroid function tests (triiodothyronine (T3): 1.75ng/ml, thyroxine (T4): 7.34ug/dl, thyroid-stimulating hormone (TSH): 4.5uIU/ml) were within normal limits. Her glycated hemoglobin (HbA1c) level was 6.5%. MRI of the brain revealed signs suggestive of benign intracranial hypertension (Figure 1A-1D). Venogram was done to rule out cerebral venous sinus thrombosis, which can lead to papilledema and signs of raised intracranial pressure, and was normal (Figure 1E-1F). Perimetry and optical coherence tomography signs were suggestive of papilledema, supporting the diagnosis of benign intracranial hypertension (Figure 2).

CSF opening pressure was 40 cmH2O. CSF drained approximately 25 ml, and the closing pressure was 19 cmH2O. The patient experienced a dramatic improvement in symptoms. CSF examination revealed a protein level of 74.2 mg/dl, glucose level of 53 (corresponding blood sugar level 82 mg/dl), and 2 lymphocytes. CSF Gram stain, culture, cartridge-based nucleic acid amplification test (CBNAAT), and cryptococcal antigen tests were all negative. The Mantoux test and QuantiFeron-TB gold assay (QIAGEN N.V., Venlo, The Netherlands) were also negative.

**Figure 3:**
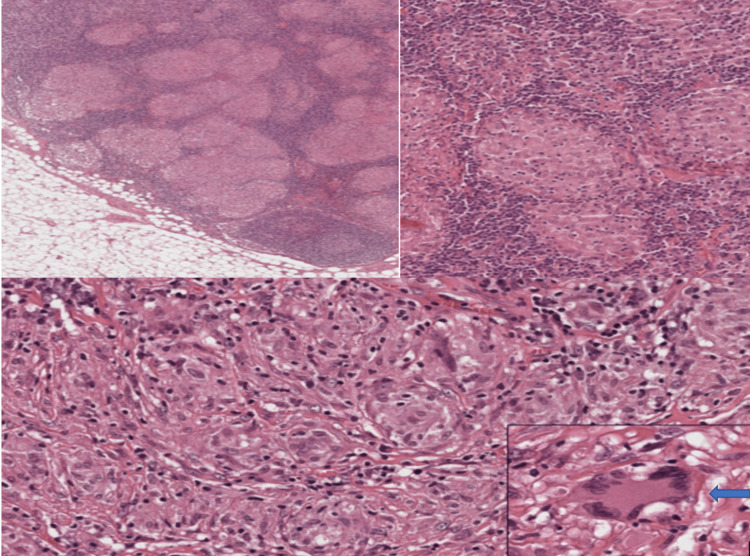
Histopathology of hilar lymph node showed non-caseating granulomas that appeared eosinophilic, composed of epithelioid histiocytes with no caseating necrosis, and giant cells were present

C-reactive protein (CRP) was 51.80, and erythrocyte sedimentation rate (ESR) was 105 mm/hour. Antinuclear antibody (ANA) by immunofluorescent method was weakly positive, but ANA blot test was negative. Antineutrophil cytoplasmic antibody (ANCA) profile was negative. Ultrasonography of the abdomen and pelvis showed normal results. Bilateral hilar lymphadenopathy was observed on high-resolution CT (HRCT) of the thorax. Positron emission tomography (PET)-CT showed metabolic activity in the bilateral hilar lymph nodes. Consequently, the patient underwent a transbronchial lymph node biopsy. Histopathology (Figure 3) was suggestive of sarcoidosis, and Ziehl-Neelsen (ZN) stain was negative.

Serum ACE level was 104.77 U/L (normal range: 16-85 U/L) and serum ionized calcium was 1.5 mmol/L (normal range: 1.16-1.31 mmol/L). The patient was started on oral steroids and acetazolamide, which resulted in significant improvement in symptoms. Currently, the patient is under regular follow-up and has not reported any new symptoms.

## Discussion

The prevalence of sarcoidosis is higher in females compared to males [7-12]. Sarcoidosis commonly manifests in the fourth decade of life [11-13], and our patient's age aligns with this pattern. Sarcoidosis affects multiple systems in the body, with respiratory system involvement being the most frequent [1-3,6]. This was demonstrated in our case with bilateral hilar lymphadenopathy. Nervous system involvement is rare in sarcoidosis, but cranial mononeuropathy, particularly involving the facial nerve, is a more common presentation of neurosarcoidosis [2,5,6]. This which was also evident in our patient with a history of Bell's palsy. To our knowledge, other reported cranial nerve involvements include the optic nerve and the VIII cranial nerve [14,15]. Granulomatous infiltration in the hypothalamus and pituitary can result in various hormonal insufficiencies [16,17]. Our patient too had hypothyroidism with positive anti-thyroid peroxidase (TPO) antibody. ACE level is a conventional marker used to assess the disease activity of sarcoidosis [18], and it was also elevated in our case.

Sarcoidosis presenting as benign intracranial hypertension is an extremely rare occurrence, with only a few reported cases till now [19,20]. The presence of biopsy-proven non-caseating granulomas support the diagnosis of sarcoidosis [21,22]. Our patient responded well to treatment with oral steroids, as per guidelines [23-25]. She is currently under regular follow-up, and there have been no new occurrences of sarcoidosis-related symptoms.

## Conclusions

The initial presentation of sarcoidosis as neurosarcoidosis poses a significant diagnostic challenge and often requires a high index of suspicion. In the case of our patient, recurrent facial nerve palsy was observed, which raised the possibility of neurosarcoidosis as one of the differential diagnoses. Although benign intracranial hypertension is an uncommon presentation of neurosarcoidosis, early diagnosis is crucial due to the favorable response of sarcoidosis to immunosuppressive agents.
